# The effects of green coffee extract supplementation on glycemic indices and lipid profile in adults: a systematic review and dose-response meta-analysis of clinical trials

**DOI:** 10.1186/s12937-020-00587-z

**Published:** 2020-07-14

**Authors:** Omid Asbaghi, Mehdi Sadeghian, Morteza Nasiri, Mahmoud Khodadost, Azad Shokri, Bahman Panahande, Aliyar Pirouzi, Omid Sadeghi

**Affiliations:** 1 Nutritional Health Research Center, Lorestan University of Medical Sciences, Khorramabad, Iran; 2 Department of Nutrition, School of Allied Medical Sciences, Ahvaz Jundishapur University of Medical Sciences, Ahvaz, Iran; 3 Student Research Committee, Shiraz University of Medical Sciences, Shiraz, Iran; 4 Department of Operating Room Nursing, School of Nursing and Midwifery, Shiraz University of Medical Sciences, Shiraz, Iran; 5 Department of Epidemiology, School of Public Health, Shahid Beheshti University of Medical Sciences, Tehran, Iran; 6 Department of Epidemiology, School of Public Health, Iran University of Medical Sciences, Tehran, Iran; 7 Social Determinants of Health Research Center, Research Institute for Health Development, Kurdistan University of Medical Sciences, Sanandaj, Iran; 8 Department of Nutrition, School of Health, Yasuj University of Medical Sciences, Yasuj, Iran; 9 Gerash University of Medical Sciences, Gerash, Iran; 10 Students’ Scientific Research Center, Tehran University of Medical Sciences, Tehran, Iran; 11 Department of Community Nutrition, School of Nutritional Sciences and Dietetics, Tehran University of Medical Sciences, Tehran, Iran

**Keywords:** Chlorogenic acid, Green coffee, Lipid profile, Glycemic indices

## Abstract

**Background:**

The role of coffee consumption in the risk of cardiovascular diseases has been debated for many years. The current study aimed to summarize earlier evidence on the effects of green coffee extract (GCE) supplementation on glycemic indices and lipid profile.

**Methods:**

We searched available online databases for relevant clinical trials published up to October 2019. All clinical trials investigating the effect of GCE supplementation, compared with a control group, on fasting blood glucose (FBG), serum insulin, total cholesterol (TC), triglyceride (TG), low-density lipoprotein (LDL), and high-density lipoprotein (HDL) were included. Overall, 14 clinical trials with a total sample size of 766 participants were included in the current meta-analysis.

**Results:**

We found a significant reducing effect of GCE supplementation on FBG (weighted mean difference (WMD): -2.35, 95% CI: − 3.78, − 0.92 mg/dL, *P* = 0.001) and serum insulin (WMD: -0.63, 95% CI: − 1.11, − 0.15 μU/L, *P* = 0.01). With regard to lipid profile, we observed a significant reduction only in serum levels of TC following GCE supplementation in the overall meta-analysis (WMD: -4.51, 95% CI: − 8.39, − 0.64, *P* = 0.02). However, subgroup analysis showed a significant reduction in serum TG in studies enrolled both genders. Also, such a significant reduction was seen in serum levels of LDL and HDL when the analyses confined to studies with intervention duration of ≥8 weeks and those included female subjects. In the non-linear dose-response analyses, we found that the effects of chlorogenic acid (CGA) dosage, the main polyphenol in GCE, on FBG, TG and HDL were in the non-linear fashions.

**Conclusion:**

In conclusion, we found that GCE supplementation improved FBG and serum levels of insulin and TC. Also, there was a significant improvement in other markers of lipid profile in some subgroups of clinical trials.

## Background

Cardiovascular diseases (CVDs) are the number one cause of death worldwide [1]. Dyslipidemia and impaired glucose tolerance (IGT) are among the primary risk factors for the development and progression of CVDs and type 2 diabetes [2, 3]. Reversal of these risk factors leads to a considerable reduction in the risk of these chronic diseases [4–7]. Although current guideline recommends dietary regulations as the first-line therapy for dyslipidemia and glucose disturbance, only a modest amelioration has been achieved using these methods [8].

Coffee is widely consumed in the world containing a range of phytochemicals. Previous studies have shown beneficial effects of coffee consumption on several health conditions including metabolic syndrome, type 2 diabetes, and vascular function [9, 10]. The most commonly found phytochemicals in the coffee are phenolic compounds, primarily chlorogenic acid (CGA) [11]. CGA is the ester of caffeic acid with quinic acid that belongs to the family of hydroxycinnamic acid [12]. Anti-lipidemic and anti-diabetic properties of CGA have been demonstrated in animal studies [13–15]. However, findings from human clinical trials are not consistent [16–29]. Green coffee extract (GCE) and its CGA showed hypolipidemic effects on serum levels of triglyceride (TG) and total cholesterol (TC) in patients with IGT [29] and those with non-alcoholic fatty liver disease [26]; however, the effects on circulating levels of high-density lipoprotein (HDL) and low-density lipoprotein (LDL) were inconsistent. Some studies showed a significant increase in serum HDL following GCE intake [17, 19], while others could not find any significant results [20, 21, 24]. Considering insulin resistance, fasting blood glucose (FBG) or serum insulin significantly reduced following GCE administration in some studies [18, 24, 29], but no significant changes were observed in some others [23, 27].

A previous meta-analysis of randomized controlled trials (RCTs) found increased levels of serum TC, LDL, and TG following coffee intake [30]; however, to our knowledge, there is no study summarizing available findings on the effects of green coffee consumption on glycemic indices and lipid profile. Therefore, we aimed to conduct a systematic review and meta-analysis to summarize current evidence on the effects of GCE supplementation on glycemic and lipid profiles in adults.

## Methods

This study was performed based on the PRISMA (Preferred Reporting Items for Systematic Reviews and Meta-Analyses) protocol for reporting systematic reviews and meta-analyses [31].

### Search strategy

A comprehensive literature search was performed using online databases of PubMed, Scopus, Web of Science and Google Scholar up to October 2019. The aim of the search was to identify clinical trials that investigated the effects of GCE supplementation on glycemic indices and lipid profile in adults. The following keywords were used in the search strategy: (“Green coffee” OR “Green coffee extract” OR “Green coffee bean extract” OR “Chlorogenic acid” OR “Chlorogenic” OR “GCR” OR “CGA”) AND (Triglyceride OR Triacylglycerol OR TG OR cholesterol OR lipoprotein OR “very low density lipoprotein” OR VLDL OR “low density lipoprotein” OR LDL OR “high density lipoprotein” OR HDL OR “lipid profile” OR “fasting blood sugar” OR glucose OR insulin OR “ glycosylated hemoglobin” OR HbA1c OR FBS OR FBG). No restriction was considered for the time and language of publications. We conducted a manual search in the reference lists of the relevant studies to avoid missing any eligible publication. Unpublished studies were not considered.

### Inclusion criteria

We included eligible studies that met the following criteria: 1) placebo-controlled clinical trials 2) those that performed on adult subjects (≥18 years old), 3) studies that administered green coffee extract in the forms of supplement or powder added to foods or beverages, 4) those that did intervention for at least 2 weeks, and 5) controlled trials that reported mean changes and SDs of glycemic indices or lipid profile throughout the trial for both the intervention and control groups or presented required information for calculation of those effect sizes. If more than one article was found for one dataset, the more complete one was selected. Clinical trials with an additional arm were considered as 2 separate studies.

### Exclusion criteria

In the current meta-analysis, we excluded:1) in vitro and animal studies, 2) studies with a cohort, cross-sectional, and case-control design, 3) review articles, 3) trials without a placebo or control group.

### Data extraction

The following information was extracted from each eligible clinical trial by two independent investigators: name of the first author, publication year, individuals’ characteristics (mean age and sex), design, sample size (control and intervention groups), type of intervention, dosage of GCE and CGA, duration of intervention, and mean changes and SDs of outcome variables throughout the trial for the intervention and control groups. When data for glycemic or lipid measures were reported in different units, we converted them to the most frequently used unit.

### Risk of bias assessment

We used the Cochrane quality assessment tool to assess the risk of bias for each study included in the current meta-analysis [32]. This tool contained seven domains including random sequence generation, allocation concealment, reporting bias, performance bias, detection bias, attrition bias, and other sources of bias. Each domain was given a “high risk” score if RCT comprised methodological defects that may have distorted the results, a “low risk” score if the defect was considered ineffectual and an “unclear risk” score if the information was not sufficient to determine the impact. If the trial had “low risk” for all domains, it was labeled as a high-quality study with a totally low risk of bias. The risk of bias assessment was done independently by two reviewers.

### Statistical analysis

Mean differences in changes of the outcome variables (FBG, insulin, TG, TC, LDL, and HDL), comparing GCE and control groups, were used to obtain the overall effect sizes. When mean changes were not reported, we computed them by considering changes in each outcome variable during the intervention. If outcome variables (FBG, TG, TC, LDL, and HDL) were reported in mmol/L, we converted them to mg/dl through available suitable formulas. We also converted standard errors (SEs), 95% confidence intervals (CIs), and interquartile ranges (IQRs) to SDs using relevant formulas [33–35]*.* To obtain the overall effect sizes, we applied a random-effects model taking between-study variations into account. Heterogeneity was determined by the I^2^ statistic and Cochrane’s Q test. I^2^ value > 50% or *P* < 0.05 for the Q-test was considered as significant between-study heterogeneity [36, 37]. To find probable sources of heterogeneity, subgroup analyses were performed according to the predefined criteria including gender (both/male/female), length of intervention (≥8/< 8 weeks), baseline levels of glycemic and lipid measures (abnormal/normal levels) and participants’ compliance (acceptable/non-acceptable or unclear). To determine the non-linear potential effects of CGA dosage (mg/d) on glycemic and lipid indices, fractional polynomial modeling was executed. Due to the lack of information on the dosage of GCE in some included studies, we decided to perform non-linear dose-response analysis for CGA dosage. Sensitivity analysis was used to explore the extent to which inferences might depend on a particular study. The possibility of publication bias was evaluated by the formal test of Begg. The meta-analysis was carried out by the use of the Stata, version 11.2 (StataCorp). *P* value *<* 0.05 was considered as significant level.

## Results

After the initial search, a total of 1571 studies were identified. After removing duplicate publications, 976 articles remained, out of which 958 studies were identified as unrelated when screening for title and abstract. After assessing the full text of remained articles, we excluded one study in which the effects of GCE in combination with olive leaf and beetroot were assessed [38]. We also excluded a study by Salamat et al. [39] that reported data only for oxidized LDL, not the natural type. Two studies that were quasi-experimental with no control group were excluded as well [40, 41]. Two RCTs were conducted on a similar dataset; however, due to assessing different outcome variables, both of them were included [21, 25]. Finally, 14 studies remained for the current systematic review and meta-analysis [16–29], out of which 11 studies presented data for FBG [16, 18, 19, 22–29], 7 studies for serum concentrations of insulin [18, 19, 24–26, 29], 13 trials for serum concentrations of TC [16–24, 26–29], and 12 trials for serum concentrations of TG, LDL, and HDL [17–24, 26–29]. Data on other glycemic indices including glycosylated hemoglobin A1c (HbA1c) (*n* = 3) and homeostatic model assessment for insulin resistance (HOMA-IR) (*n* = 2) were not sufficient for the meta-analysis. Flow diagram of study selection is presented in Supplemental Figure 1.

### Findings from the systematic review

Characteristics of the 14 clinical trials included in the current systematic review are illustrated in Table 1. The total sample size of the selected studies was 766 adult participants including 380 subjects in the GCE group and 386 subjects in the control group. Studies were published from 2004 to 2019. Out of 14 included studies, 3 were performed in western countries [21, 25, 29] and others were conducted in Asia [16–20, 22–24, 26–28]. The dosage of GCE among the included studies was between 90 and 6000 mg/day. Also, the dosage of CGA, as the main polyphenol in GCE, was between 13.5 and 1200 mg/day. The duration of intervention varied from 2 to 16 weeks.

**Table 1:** Characteristics of included studies investigating the effects of GCE supplementation on glycemic and lipid measures

Author, year	Design	Participants, n	Health condition	Age, year^**1**^	Intervention		Duration (week)	Outcomes (changes)^**1**^		Adjust/matching
					Treatment group	Control group		Treatment group	Control group	
Park et al. 2010 [23]	RA/DB/ Parallel	F: 43, Int: 23, Con: 20	Overweight/ obesity	Int: 33.1 ± 9.20 Con: 33.1 ± 9.79	200 mg/d GCE containing 56.8 mg CGA	Placebo	8	TG: −19.82 ± 43.73 mg/dL TC: − 1.65 ± 16.64 mg/dL LDL: − 1.26 ± 15.10 mg/dL HDL: − 2.35 ± 5.27 mg/dL FBG: − 1.78 ± 7.91 mg/dL Insulin: − 0.87 ± 3.88 μU/L	TG: − 3.30 ± 33.18 mg/dL TC: − 1.55 ± 38.10 mg/dL LDL: − 1.05 ± 37.65 mg/dL HDL: − 1.95 ± 7.55 mg/dL FBG: − 2.20 ± 6.30 mg/dL Insulin: − 1.11 ± 3.44 μU/L	No
Haidari et al. 2017 [19]	RA/DB/ Parallel	F: 64, Int: 30, Con: 34	Obesity	Int: 20–45 Con: 20–45	400 mg/d GCE containing 180 mg CGA	Placebo	8	TG: −4.0 ± 20.79 mg/dL TC: − 12.0 ± 8.46 mg/dL LDL: − 11.0 ± 8.11 mg/dL HDL: 1.0 ± 1.00 mg/dL FBG: − 0.9 ± 3.61 mg/dL Insulin: − 0.1 ± 0.42 μU/L	TG: −5.0 ± 16.53 mg/dL TC: − 5.0 ± 8.29 mg/dL LDL: − 2.0 ± 5.55 mg/dL HDL: − 2.0 ± 0.90 mg/dL FBG: − 0.34 ± 3.42 mg/dL Insulin: 0.20 ± 0.44 μU/L	Fat mass, fiber and energy intake, physical activity
Shahmohammadi et al. 2017 [26]	RA/DB/ Parallel	F/M: 44, Int: 22, Con: 22	Overweight and NAFLD	Int: 41.36 ± 7.69 Con: 44.50 ± 5.24	1000 mg/d GCE containing 500 mg CGA	Placebo	8	TG: − 37.77 ± 31.62 mg/dL TC: − 16.96 ± 20.10 mg/dL LDL: − 2.41 ± 14.73 mg/dL HDL: − 0.36 ± 7.53 mg/dL FBG: − 6.91 ± 5.27 mg/dL Insulin: − 0.87 ± 1.85 μU/L	TG: − 2.41 ± 34.83 mg/dL TC: 0.55 ± 20.87 mg/dL LDL: − 5.72 ± 12.08 mg/dL HDL: 0.31 ± 4.00 mg/dL FBG: − 2.36 ± 8.57 mg/dL Insulin: − 0.15 ± 1.50 μU/L	No
Roshan et al. 2018 [24]	RA/DB/ Parallel	F/M: 43, Int: 21, Con: 22	Metabolic syndrome	Int: 52.76 ± 9.83, Con: 51.95 ± 8.67	800 mg/d GCE containing 368 mg CGA	Placebo	8	TG: − 6.19 ± 53.09 mg/dL TC: 1.54 ± 39.38 mg/dL LDL: 3.86 ± 18.14 mg/dL HDL: 1.93 ± 8.49 mg/dL FBG: − 5.04 ± 60.18 mg/dL Insulin: − 2.82 ± 4.2 μU/L	TG: − 22.12 ± 76.99 mg/dL TC: 1.54 ± 27.79 mg/dL LDL: 3.86 ± 29.72 mg/dL HDL: 1.93 ± 3.47 mg/dL FBG: 29.36 ± 40 mg/dL Insulin: − 0.39 ± 6.46 μU/L	Sex
Zuniga et al. 2018 [29]	RA/DB/ Parallel	F/M: 26, Int: 12, Con: 14	IGT	30–60	1200 mg/d CGA	Placebo	12	TG: − 26.55 ± 31.90 mg/dL TC: − 7.72 ± 13.91 mg/dL LDL: − 15.45 ± 25.9 mg/dL HDL: 3.86 ± 9.29 mg/dL FBG: − 3.61 ± 4.55 mg/dL	TG: 8.85 ± 39.17 mg/dL TC: 15.45 ± 23.48 mg/dL LDL: 3.86 ± 25.66 mg/dL HDL: 0 ± 9.29 mg/dL FBG: 1.8 ± 4.33 mg/dL	No
Aghaei et al. 2018 [17]	RA/SB/Cross-over	F: 30, Int: 15, Con: 15	Overweight	Int: 30.4 ± 26.82, Con: 28.5 ± 20.53	Training plus 90 mg/d GCE added to a 250 mL water	Training	8	TG: − 10.8 ± 25.56 mg/dL TC: − 6.67 ± 17.12 mg/dL LDL: − 3.7 ± 12.55 mg/dL HDL: 4.1 ± 4.66 mg/dL	TG: − 59.44 ± 45.60 mg/dL TC: − 21.8 ± 27.47 mg/dL LDL: − 9.26 ± 17.11 mg/dL HDL: 0.06 ± 5.59 mg/dL	No
Kim et al. 2012	RA/DB/ Parallel	F: 20, Int: 10, Con: 10	Obesity	> 18	100 mg/d GCE containing 29.4 mg CGA	Placebo	8	TC: − 2.5 ± 12.22 mg/dL FBG: − 2.8 ± 3.75 mg/dL	TC: −2.4 ± 32.47 mg/dL FBG: 2.7 ± 4.28 mg/dL	No
Kozuma et al. 2005 [20]	RA/DB/ Parallel	M: 117, Int 1: 29, Int 2: 28, Int 3: 31, Con: 29	Hypertension	Int 1: 42.9 ± 8.2, Int 2: 43.3 ± 8.3, Int 3: 43.4 ± 8.4, Con: 43.1 ± 9.1	Int 1: 46 mg/d GCE containing 25 mg CGA Int 2: 93 mg/d GCE containing 50 mg CGA Int 3: 185 mg/d GCE containing 100 mg CGA GCE was added to a cup of hot water	Control drink	4	Int 1: TG: 1.3 ± 40.3 mg/dL TC: 4.3 ± 19.04 mg/dL LDL: 3 ± 18.02 mg/dL HDL: 0 ± 11.0 mg/dL Int 2: TG: 2.3 ± 46.9 mg/dL TC: − 7.1 ± 19.73 mg/dL LDL: − 4.6 ± 22.13 mg/dL HDL: 0 ± 8.34 mg/dL Int 3: TG: 6.2 ± 38.9 mg/dL TC: − 7.3 ± 17.09 mg/dL LDL: − 8.2 ± 19.07 mg/dL HDL: 0 ± 8.98 mg/dL	TG: 4.7 ± 38.33 mg/dL TC: − 1.6 ± 19.73 mg/dL LDL: − 4.5 ± 19.83 mg/dL HDL: 0 ± 7.46 mg/dL	Blood pressure
Watanabe et al. 2006 [28]	RA/DB/ Parallel	M/F: 28, Int: 14, Con: 14	Hypertension	Int: 52 ± 11, Con: 51 ± 8	480 mg/d GCE containing 140 mg CGA in the form of a drink	Placebo drink	12	TG: 4.0 ± 61.51 mg/dL TC: − 3.0 ± 22.75 mg/dL LDL: 3.0 ± 16.56 mg/dL HDL: 1.0 ± 11.22 mg/dL FBG: 1.0 ± 13.48 mg/dL	TG: − 4.0 ± 112.30 mg/dL TC: 12.0 ± 27.43 mg/dL LDL: 14.0 ± 22.75 mg/dL HDL: 6.0 ± 15.78 mg/dL FBG: 1.0 ± 24.87 mg/dL	No
Lopez et al. 2019 [21]	RA/SB/Cross-over	M/F: 52,: Int: 26, Con: 26	Normocholesterolemics (*n* = 25)	18–45	Daily intake of roasted/unroasted green coffee beverage containing 445.2 mg CGA	Control drink	8	TG: −0.3 ± 21.30 mg/dL TC: 2.2 ± 14.08 mg/dL LDL: 0.5 ± 14.10 mg/dL HDL: 2.3 ± 8.90 mg/dL	TG: − 1.3 ± 21.43 mg/dL TC: 1.2 ± 14.57 mg/dL LDL: − 0.2 ± 12.18 mg/dL HDL: 0.8 ± 8.70 mg/dL	No
			Hypercholesterolemics (*n* = 27)					TG: − 20.4 ± 23.38 mg/dL TC: − 21.0 ± 15.94 mg/dL LDL: − 18.8 ± 15.90 mg/dL HDL: − 0.4 ± 9.90 mg/dL	TG: − 16.7 ± 23.52 mg/dL TC: − 14.3 ± 15.51 mg/dL LDL: − 15.3 ± 13.83 mg/dL HDL: − 3.7 ± 9.66 mg/dL	
Suzuki et al. 2019 [27]	DB/Parallel	M: 16, Int: 8, Con: 8	Healthy	Int: 44.6 ± 6.2 Con: 44.2 ± 5.4	Daily intake of GCE containing 300 mg CGA in the form of a drink	Control drink	2	TG: 3.7 ± 24.82 mg/dL TC: 2.4 ± 22.32 mg/dL LDL: 3.0 ± 18.72 mg/dL HDL: 0.5 ± 7.08 mg/dL FBG: − 0.7 ± 3.16 mg/dL	TG: 4.5 ± 25.20 mg/dL TC: − 3.4 ± 14.80 mg/dL LDL: − 1.1 ± 12.91 mg/dL HDL: − 0.7 ± 4.64 mg/dL FBG: 0.7 ± 2.84 mg/dL	No
Fukagawa et al. 2017 [18]	RA/DB/ Parallel	F: 49, Int: 23 Con: 26	Mildly xerotic skin	25–40	Daily intake of GCE containing 270 mg CGA in the form of a drink	Control drink	8	TG: 3.6 ± 28.13 mg/dL TC: − 2.6 ± 13.98 mg/dL LDL: − 1.4 ± 15.57 mg/dL HDL: − 2.2 ± 10.07 mg/dL FBG: 0.4 ± 4.89 mg/dL Insulin: 0.04 ± 3.16 μU/L	TG: 9.9 ± 77.97 mg/dL TC: 2.9 ± 21.25 mg/dL LDL: 1.9 ± 18.05 mg/dL HDL: 0.8 ± 8.87 mg/dL FBG: 0.7 ± 6.94 mg/dL Insulin: 3.44 ± 6.97 μU/L	No
Sarria et al. 2018 [25]	RA/SB/Cross-over	M/F: 52,: Int: 26, Con: 26	Normocholesterolemics (*n* = 25)	18–45	Daily intake of roasted/unroasted green coffee beverage containing 445.2 mg CGA	Control drink	8	FBG: −3.0 ± 4.92 mg/dL Insulin: − 0.05 ± 1.20 μU/L	FBG: 0.9 ± 4.60 mg/dL Insulin: 1.08 ± 1.17 μU/L	No
			Hypercholesterolemics (*n* = 27)					FBG: − 3.75 ± 5.44 mg/dL Insulin: − 0.39 ± 1.43 μU/L	FBG: − 2.38 ± 5.02 mg/dL Insulin: − 0.15 ± 1.39 μU/L	
Ochiai et al. 2004 [22]	Parallel	M: 20, Int: 10, Con: 10	Healthy	> 18	Daily intake of a beverage containing GCE and 140 mg CGA	Control drink	16	TG: 7.8 ± 144.45 mg/dL TC: − 3.7 ± 27.53 mg/dL LDL: 0.5 ± 20.12 mg/dL HDL: − 3.3 ± 9.31 mg/dL FBG: − 4.2 ± 6.64 mg/dL	TG: − 5.2 ± 81.38 mg/dL TC: 2.3 ± 20.17 mg/dL LDL: 8.9 ± 15.01 mg/dL HDL: − 1.7 ± 9.49 mg/dL FBG: − 3.1 ± 4.75 mg/dL	No

^1^Values are mean ± SD or range (for age)

*Abbreviations*: *GCE* green coffee extract, *CGA* chlorogenic acid, *M* male, *F* female, *RA* randomized, *DB* double-blinded, *SB* single-blinded, *Con* control, *TG* triglycerides, *TC* total cholesterol, *LDL* low-density lipoprotein, *HDL* high-density lipoprotein, *FBG* fasting blood glucose, *NAFLD* non-alcoholic fatty liver disease, *IGT* impaired glucose tolerance

Of fourteen studies, six trials administered GCE in the form of supplement [16, 19, 23, 24, 26, 29], while eight studies administered in the form of powder added to boiled water or other beverages [17, 18, 20–22, 25, 27, 28]. Out of fourteen studies, two were performed on healthy individuals [22, 27] and others were conducted on subjects with overweight or obesity, dyslipidemia, hypertension, metabolic syndrome, non-alcoholic fatty liver disease, impaired glucose tolerance and mildly xerotic skin. According to the Cochrane Risk of Bias Assessment tool, none of the clinical trials had a low risk of bias in all domains of this tool (Supplemental Table 1).

Among eleven studies on FBG, four trials reported a lowering effect of GCE supplementation on FBG levels [16, 24–26, 29] and the other trials revealed no significant effect. Only in two studies, GCE supplementation could reduce serum levels of insulin [19, 25] while the other trials could not find any significant change. There were 12 clinical trials on serum concentrations of TG, from which two studies revealed a lowering effect [26, 29], one study showed an increasing effect [17], and the others indicated no significant effect on serum TG concentrations following GCE supplementation. Out of thirteen studies on TC, 3 studies found a significant reduction in serum TC concentrations after supplementation with GCE [19, 26, 29]. Only one study revealed a reducing effect of GCE supplementation on serum concentrations of LDL [19] while others did not reach statistical significance. Among twelve studies on serum HDL levels, only two studies showed an increasing effect of GCE supplementation on serum concentrations of HDL [17, 19] whereas others failed to find any significant effect.

### Findings from the meta-analysis

All fourteen clinical trials were included in the meta-analysis. These studies included 766 participants aged 18 years and over. There was a trial with three intervention arms with different dosages of GCE compared with one control group [20]. This study was considered as three separate studies. In two studies, findings were reported separately for normocholesterolemic and hypercholesterolemic participants [21, 25]. Therefore, we considered each study as two separate studies.

#### The effect of GCE on FBG levels

**Fig. 1:**
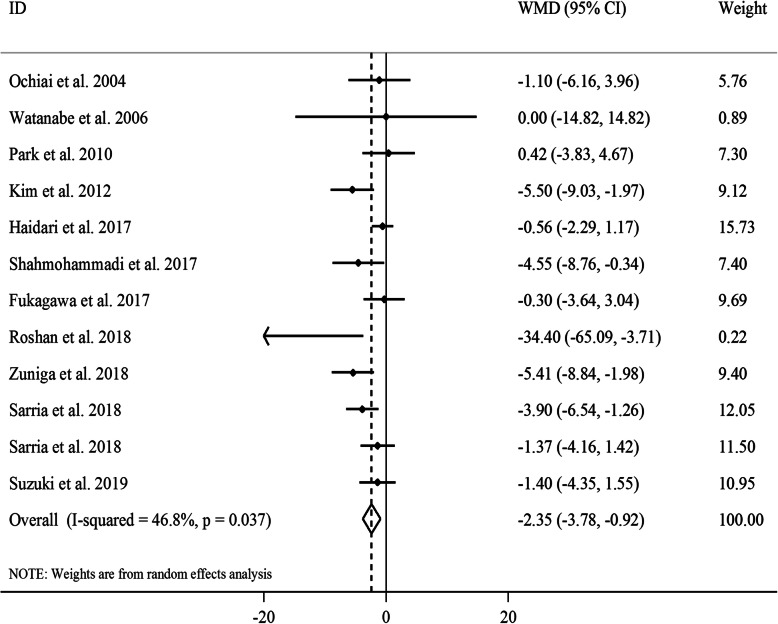
Forest plot for the effect of GCE supplementation on FBG levels, expressed as mean differences between intervention and control groups. Horizontal lines represent 95% CIs. Diamonds represent pooled estimates from random-effects analysis. GCE: green coffee extract, FBG: fasting blood glucose, CI: confidence interval

Overall, 12 effect sizes from 11 clinical trials with a total sample size of 457 participants were included in the analysis [16, 18, 19, 22–29]. After combining effect sizes, we found a significant reducing effect of GCE supplementation on FBG levels (WMD: -2.35, 95% CI: − 3.78, − 0.92 mg/dL, *P* = 0.001) (Fig. 1). However, there was a moderate between-study heterogeneity (*I*^*2*^: 46.8, *P* = 0.03). To detect the sources of between-study heterogeneity, we performed subgroup analyses according to gender (male, female, or both), length of intervention (≥8 vs. < 8 weeks), baseline levels of FBG (≥100 vs. < 100 mg/dL) and participants’ compliance (acceptable, non-acceptable, or unclear) (Table 2). From these analyses, we found that gender, baseline levels of FBG and participants’ compliance could explain between-study heterogeneity. In addition, GCE supplementation had a reducing effect on FBG in studies performed on both genders, those with an intervention duration of ≥8 weeks, and those in which participants had a good adherence to the intervention. Findings from the sensitivity analysis revealed that the overall estimate did not depend on a particular study. Based on the Begg’s test and visual inspection of funnel plot (Supplemental Figure 2A), no evidence of a publication bias was found (*P* = 0.21).

**Table 2:** Subgroup analysis on the effects of GCE supplementation on glycemic and lipid measures

	Effect size, n	WMD^1^	95% CI^1^	*P*-value^2^	Heterogeneity	
					I^2^ (%)^3^	P-heterogeneity^4^
**The effect of GCE on FBS**						
Overall effect size	12	-2.35	−3.78, −0.92	0.001	45.8	0.037
Participants’ gender						
Male	2	−1.32	−3.87, 1.22	0.31	0.0	0.92
Female	4	−1.13	−2.47, 0.21	0.098	57.5	0.07
Both genders	6	−3.55	−5.10, −2.01	< 0.001	36.3	0.17
Intervention duration (week)						
≥ 8	11	−2.13	−3.12, −1.13	< 0.001	51.1	0.025
< 8	1	−1.40	−4.35, 1.55	0.35	–	–
Baseline FBS						
Elevated (≥100 mg/dL)	3	−5.28	−7.93, −2.63	< 0.001	44.10	0.17
Normal (< 100 mg/dL)	9	−1.59	−2.59, −0.58	0.002	24.20	0.23
Compliance						
Acceptable	5	−3.59	−5.15, −2.04	< 0.001	47.6	0.11
Unacceptable/Unclear	7	−1.16	−2.34, 0.02	0.053	15.5	0.31
**The effect of GCE on insulin levels**						
Overall effect size	7	−0.63	−1.11, − 0.15	0.01	49.4	0.07
Participants’ gender						
Female	3	−0.31	−0.52, − 0.10	0.004	54.4	0.11
Both genders	4	−0.77	−1.21, − 0.33	0.001	26.0	0.26
Compliance						
Acceptable	4	−0.77	−1.21, − 0.33	0.001	26.0	0.26
Unacceptable/Unclear	3	−0.31	−0.52, − 0.10	0.004	54.4	0.11
**The effect of GCE on TG levels**						
Overall effect size	15	−3.17	−11.82, 5.49	0.47	59.1	0.002
Participants’ gender						
Male	5	−1.05	−11.70, 9.60	0.85	0.0	0.99
Female	4	2.76	−5.18, 10.70	0.49	79.6	0.002
Both genders	6	−7.83	−15.22, −0.44	0.04	68.0	0.008
Intervention duration (week)						
≥ 8	11	−2.87	−8.27, 2.53	0.29	70.6	< 0.001
< 8	4	−1.21	−11.91, 9.50	0.83	0.0	0.99
Baseline TG						
Elevated (≥150 mg/dL)	4	−4.58	−12.77, 3.60	0.27	74.9	0.008
Normal (< 150 mg/dL)	11	−1.44	−7.41, 4.53	0.64	54.4	0.02
Compliance						
Acceptable	8	−6.14	−12.44, 0.17	0.06	57.3	0.02
Unacceptable/Unclear	7	2.55	−4.93, 10.03	0.50	59.5	0.02
**The effect of GCE on TC levels**						
Overall effect size	16	−4.51	−8.39, −0.64	0.02	44.1	0.03
Participants’ gender						
Male	5	−1.51	−6.78, 3.75	0.57	5.6	0.38
Female	5	−5.29	−8.87, −1.71	0.004	44.2	0.13
Both genders	6	−7.48	−12.11, −2.86	0.002	60.4	0.03
Intervention duration (week)						
≥ 8	12	−6.11	−8.91, −3.30	< 0.001	45.9	0.04
< 8	4	−1.22	−6.65, 4.22	0.66	26.0	0.26
Baseline TC						
Elevated (≥200 mg/dL)	8	−6.06	−9.01, − 3.12	< 0.001	26.2	0.22
Normal (< 200 mg/dL)	8	−2.60	−7.28, 2.09	0.28	55.8	0.03
Compliance						
Acceptable	8	−4.88	−8.53, −1.23	0.009	59.4	0.02
Unacceptable/Unclear	8	−5.26	−8.66, −1.85	0.003	27.0	0.21
**The effect of GCE on LDL levels**						
Overall effect size	15	−2.02	−5.58, 1.54	0.27	47.6	0.02
Participants’ gender						
Male	5	0.58	−4.59, 5.76	0.83	3.4	0.39
Female	4	−6.96	−10.01, −3.90	< 0.001	61.1	0.05
Both genders	6	−1.44	−5.45, 2.57	0.48	25.5	0.24
Intervention duration (week)						
≥ 8	11	−5.01	−7.42, −2.61	< 0.001	47.9	0.04
< 8	4	1.70	−3.79, 7.19	0.54	0.0	0.44
Baseline LDL						
Elevated (≥130 mg/dL)	2	0.10	−5.73, 5.53	0.97	28.9	0.24
Normal (< 130 mg/dL)	13	−4.63	−7.02, −2.24	< 0.001	48.3	0.03
Compliance						
Acceptable	8	0.01	−3.38, 3.41	0.99	11.1	0.34
Unacceptable/Unclear	7	−6.79	−9.68, −3.90	< 0.001	39.4	0.13
**The effect of GCE on HDL levels**						
Overall effect size	15	1.08	−0.22, 2.38	0.10	37.6	0.07
Participants’ gender						
Male	5	0.06	−2.16, 2.27	0.96	0.0	0.99
Female	4	2.92	2.46, 3.39	< 0.001	62.0	0.05
Both genders	6	0.63	−1.37, 2.62	0.54	0.0	0.59
Intervention duration (week)						
≥ 8	11	2.79	2.34, 3.24	< 0.001	43.0	0.06
< 8	4	0.18	−2.12, 2.49	0.88	0.0	0.99
Baseline HDL						
Low (< 40 mg/dL)	5	2.87	2.41, 3.33	< 0.001	56.4	0.06
Normal (≥40 mg/dL)	10	0.34	−1.36, 2.04	0.69	0.0	0.81
Compliance						
Acceptable	8	0.51	−1.06, 2.09	0.52	0.0	0.91
Unacceptable/Unclear	7	2.88	2.42, 3.14	< 0.001	48.7	0.07

^1^Obtained from the fixed-effects model

^2^Refers to the mean (95% CI)

^3^Inconsistency, percentage of variation across studies due to heterogeneity

^4^Obtained from the Q-test

*Abbreviations*: *GCE* green coffee extract, *WMD* weighted mean difference, *CI* confidence interval, *TG* triglycerides, *TC* total cholesterol, *LDL* low-density lipoprotein, *HDL* high-density lipoprotein, *FBG* fasting blood glucose

In the non-linear dose-response analysis, we found that the association between dosage of CGA and FBG levels was in a non-linear fashion (Supplemental Figure 3A); such that greater reducing effect of CGA on FBG levels was seen at the dosage of ≥200 mg/day (P_non-linearity_ = 0.03).

#### The effect of GCE on serum concentrations of insulin

**Fig. 2:**
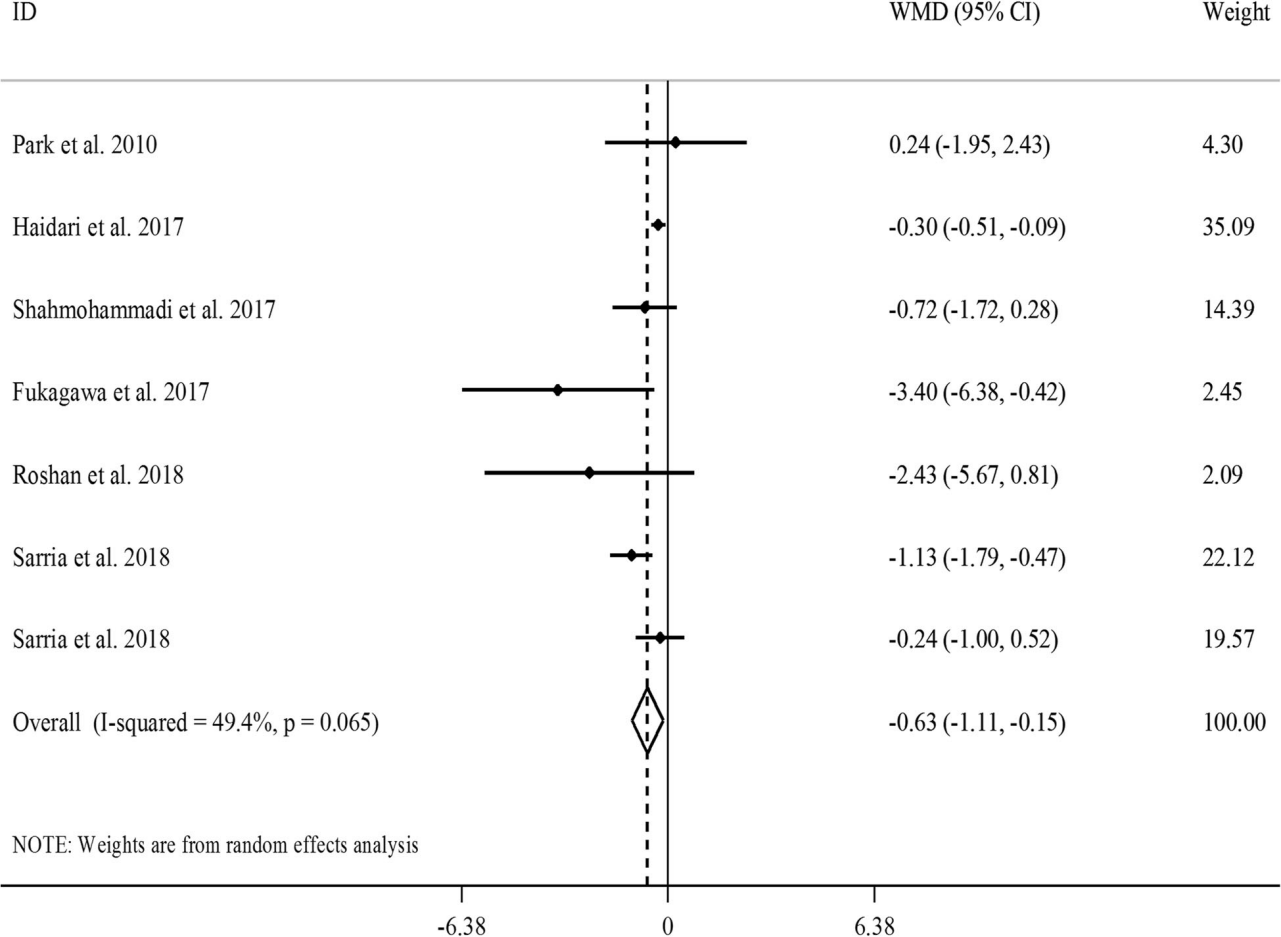
Forest plot for the effect of GCE supplementation on serum insulin concentrations, expressed as mean differences between intervention and control groups. Horizontal lines represent 95% CIs. Diamonds represent pooled estimates from random-effects analysis. GCE: green coffee extract, WMD: weighted mean difference, CI: confidence interval

Combining 7 effect sizes from 6 studies [18, 19, 24–26, 29] that included 347 participants revealed that GCE supplementation resulted in a significant reduction in serum concentrations of insulin (WMD: -0.63, 95% CI: − 1.11, − 0.15 μU/L, *P* = 0.01) (Fig. 2). We observed a moderate between-study heterogeneity (*I*^*2*^: 49.4, *P* = 0.06). In the subgroup analyses, we observed that between-study heterogeneity could be explained by gender and participants’ compliance. A significant reducing effect of GCE supplementation on serum insulin concentrations was also seen in all subgroups. Based on the sensitivity analysis, no single study influenced the overall estimate. Also, no evidence of a substantial publication bias was found according to the Begg’s test (*P* = 0.45) and funnel plot (Supplemental Figure 2B).

Based on the non-linear dose-response analysis, no significant effect of CGA dosage on serum insulin concentrations was seen (P_non-linearity_ = 0.62) (Supplemental Figure 3B).

#### The effect of GCE on serum concentrations of TG

**Fig. 3:**
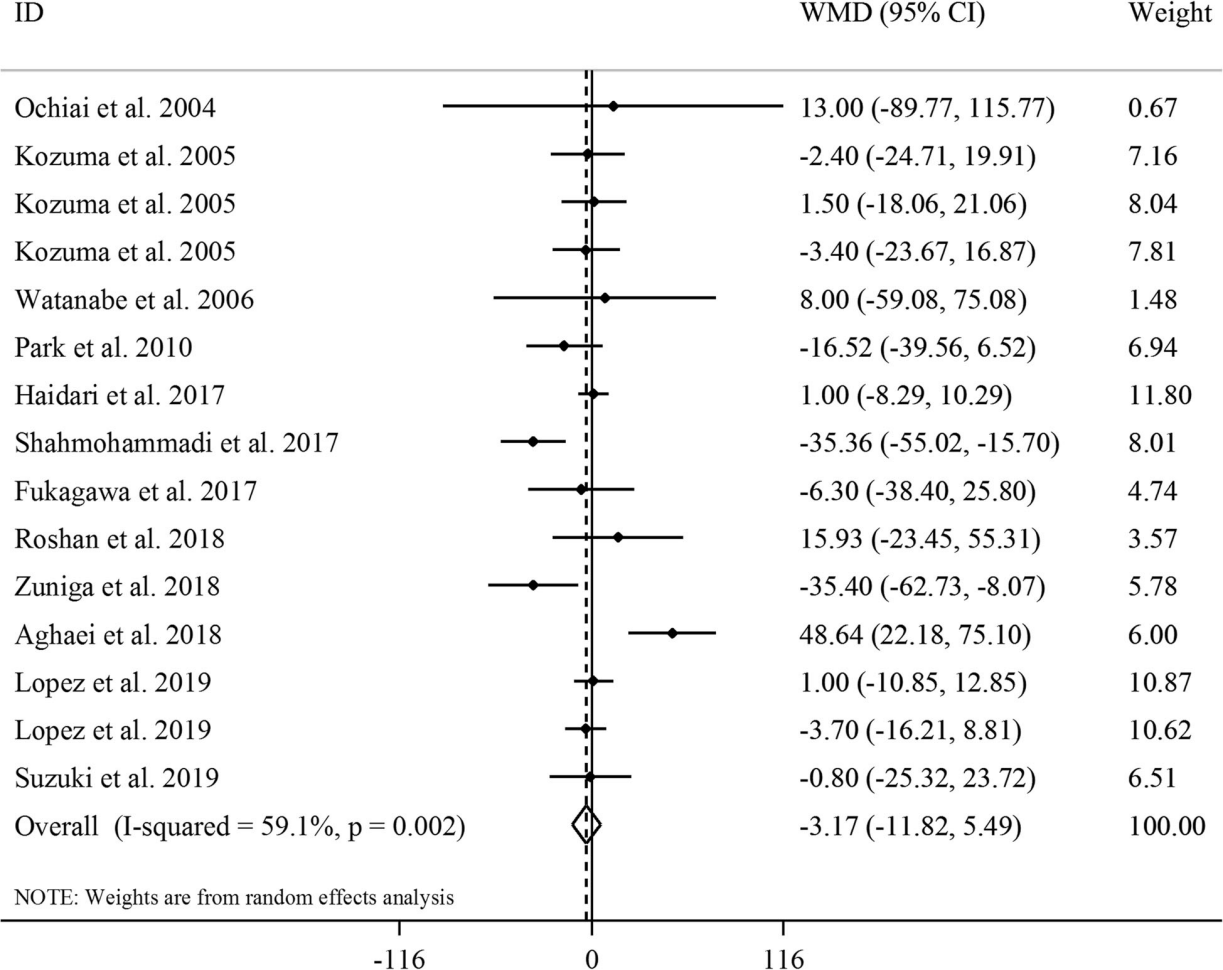
Forest plot for the effect of GCE supplementation on serum TG concentrations, expressed as mean differences between intervention and control groups. Horizontal lines represent 95% CIs. Diamonds represent pooled estimates from random-effects analysis. GCE: green coffee extract, TG: triglyceride, WMD: weighted mean difference, CI: confidence interval

Totally, 12 studies with a total sample size of 642 participants were included for this effect [17–24, 26–29]. Combining 15 effect sizes from these studies revealed no significant effect of GCE supplementation on serum TG concentrations (WMD: -3.17, 95% CI: − 11.82, 5.49 mg/dL, *P* = 0.47) (Fig. 3). However, between-study heterogeneity was significant (*I*^*2*^: 59.1, *P* = 0.002). Subgroup analyses based on gender and length of follow-up could decrease the between-study heterogeneity. In addition, we found a significant lowering effect of GCE supplementation on serum concentrations of TG in studies performed on both genders. The sensitivity analysis revealed that the exclusion of any particular study did not change the overall estimate. We found no evidence of a substantial publication bias according to the Begg’s test (*P* = 0.96) and funnel plot (Supplemental Figure 2C).

In the dose-response analysis, we found a significant non-linear association between CGA dosage and serum TG concentrations; such that dosage of CGA from low levels to 500 mg/day had a significant lowering effect on TG levels (P_non-linearity_ = 0.01), while this beneficial effect was reduced from dosage of 500 mg/day to higher amounts (Supplemental Figure 4A).

#### The effect of GCE on serum concentrations of TC

**Fig. 4:**
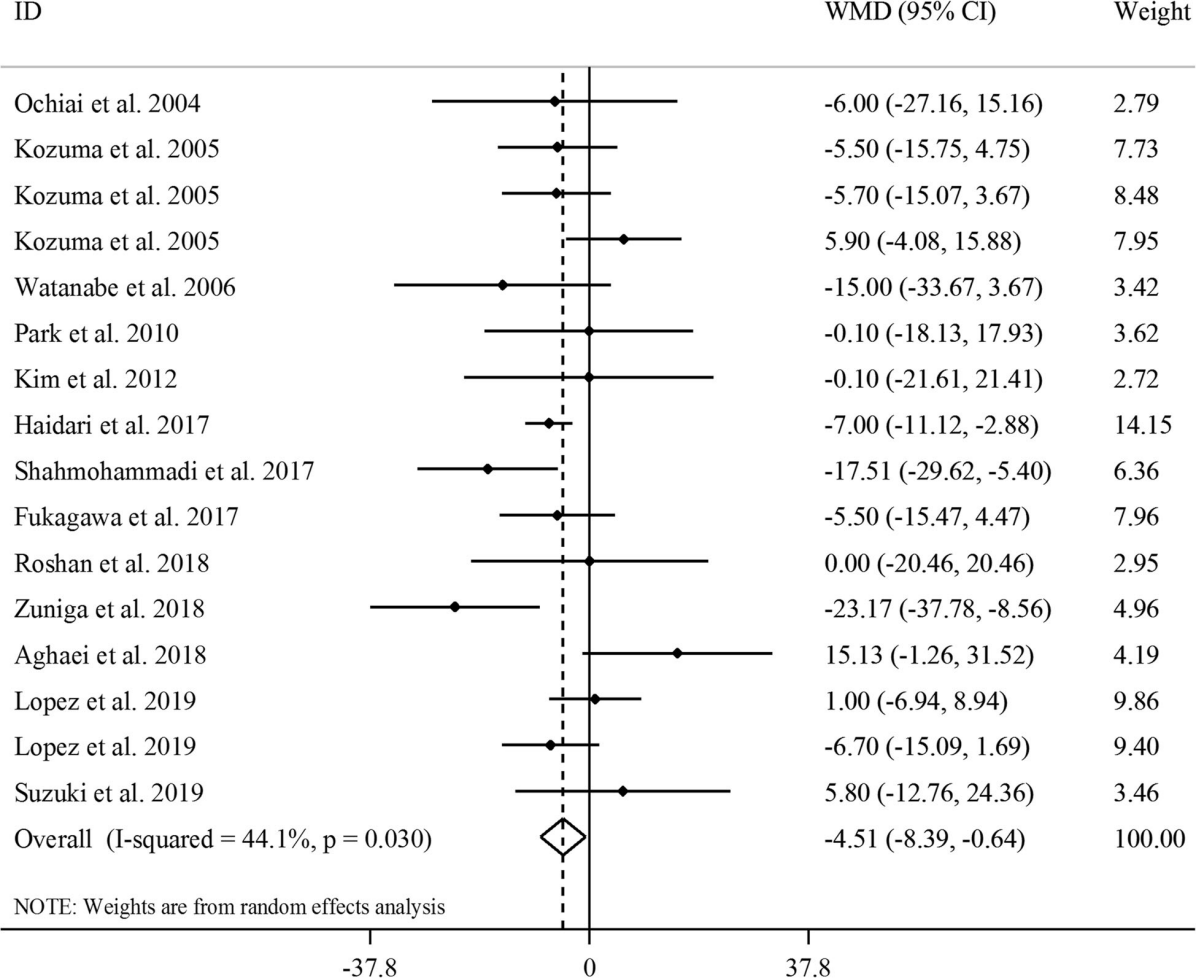
Forest plot for the effect of GCE supplementation on serum TC concentrations, expressed as mean differences between intervention and control groups. Horizontal lines represent 95% CIs. Diamonds represent pooled estimates from random-effects analysis. GCE: green coffee extract, TC: total cholesterol, WMD: weighted mean difference, CI: confidence interval

Overall, 16 effect sizes from 13 clinical trials [16–24, 26–29] with a sample size of 662 participants were included in the analysis. Combining these effect sizes, a significant reduction was seen in serum concentrations of TC following GCE supplementation (WMD: -4.51, 95% CI: − 8.39, − 0.64 mg/dL, *P* = 0.02) (Fig. 4**)**. There was an evidence of moderate between-study heterogeneity (*I*^*2*^: 44.1, *P* = 0.03). In the subgroup analyses, we found that gender, length of duration, baseline levels of TC and participants’ compliance could explain between-study heterogeneity. Based on these analyses, the effect of GCE supplementation on serum TC concentrations strengthened in studies performed on females and both genders, those with ≥8 weeks’ duration of follow-up and studies that were performed on participants with elevated baseline serum levels of TC (≥200 mg/dL). In the sensitivity analysis, significant association attenuated after exclusion of the study by Heidari et al. [19] (WMD: -4.11, 95% CI: − 8.61, 0.40 mg/dL, *P* = 0.07) and the study by Shahmohammadi et al. [26] (WMD: -3.69, 95% CI: − 7.47, 0.08 mg/dL, *P* = 0.05). However, this effect was marginally significant. Visual inspection of funnel plot (Supplemental Figure 2D) and findings from the Begg’s test revealed no evidence of a substantial publication bias (*P* = 0.58).

Based on the dose-response analysis, no significant non-linear association was observed between CGA dosage and serum TC concentrations (P_non-linearity_ = 0.15) (Supplemental Figure 4B).

#### The effect of GCE on serum concentrations of LDL

**Fig. 5:**
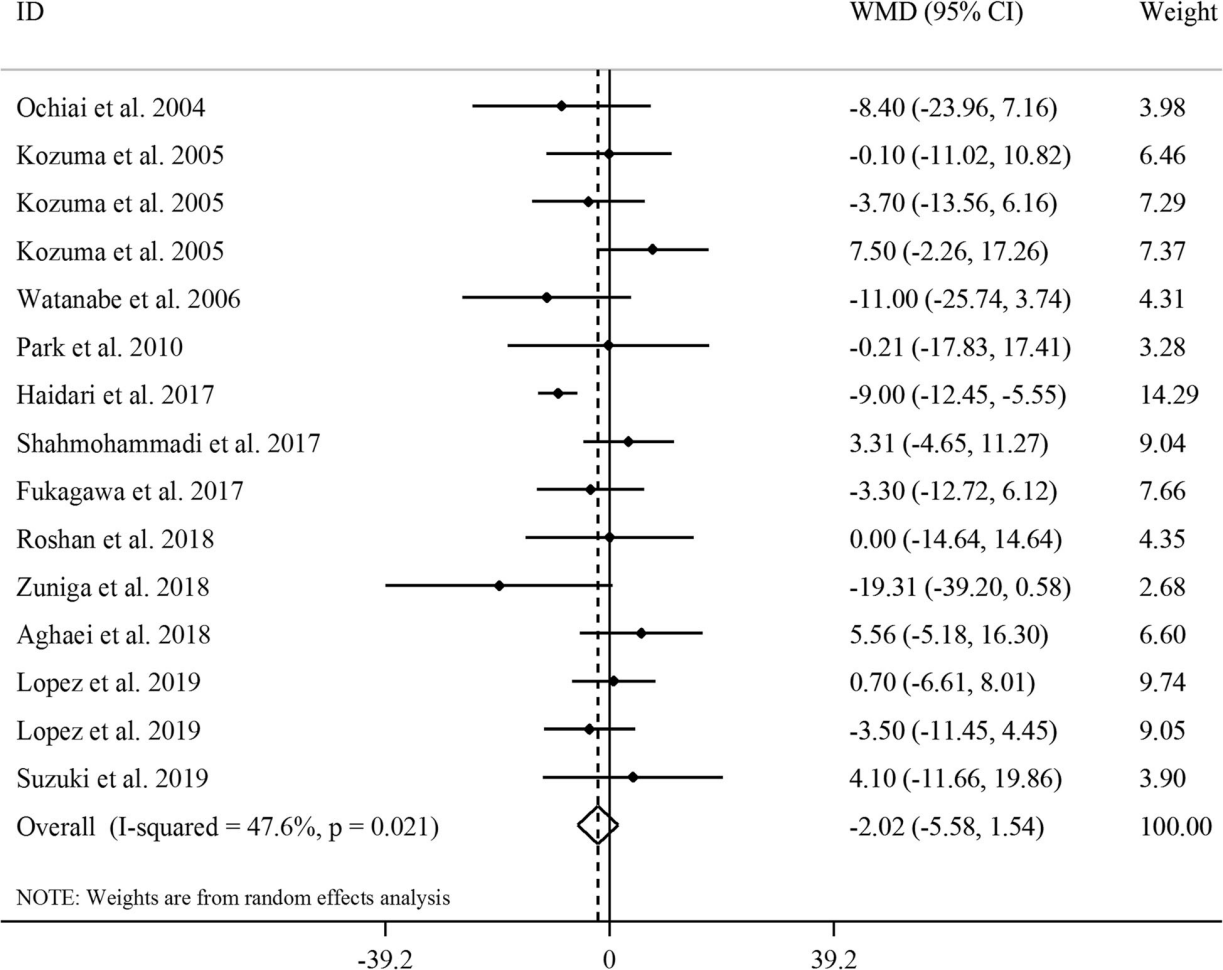
Forest plot for the effect of GCE supplementation on serum LDL concentrations, expressed as mean differences between intervention and control groups. Horizontal lines represent 95% CIs. Diamonds represent pooled estimates from random-effects analysis. GCE: green coffee extract, LDL: low-density lipoprotein, WMD: weighted mean difference, CI: confidence interval

Considering 15 effect sizes from 12 studies [17–24, 26–29] which included 642 participants, no significant effect of GCE supplementation on serum concentrations of LDL was found (WMD: -2.02, 95% CI: − 5.58, 1.54 mg/dL, *P* = 0.26, *I*^*2*^: 47.6, *P* = 0.02) (Fig. 5). Subgroup analyses based on gender, duration of follow-up, baseline values of LDL, and participants’ compliance could decrease between-study heterogeneity. From these analyses, we found that GCE supplementation significantly reduced serum LDL concentrations in studies included only female subjects, studies with a follow-up duration of ≥8 weeks, studies that included participants with normal baseline levels of LDL, and those with low or unclear compliance of participants. A sensitivity analysis revealed that the overall effect size was not influenced by a single study. No evidence of a substantial publication bias was seen based on the results from the Begg’s test (*P* = 0.40) and funnel plot (Supplemental Figure 2E).

In the dose-response analysis, no significant association was found between CGA dosage and serum levels of LDL (P_non-linearity_ = 0.27) (Supplemental Figure 4C).

#### The effect of GCE on serum concentrations of HDL

**Fig. 6:**
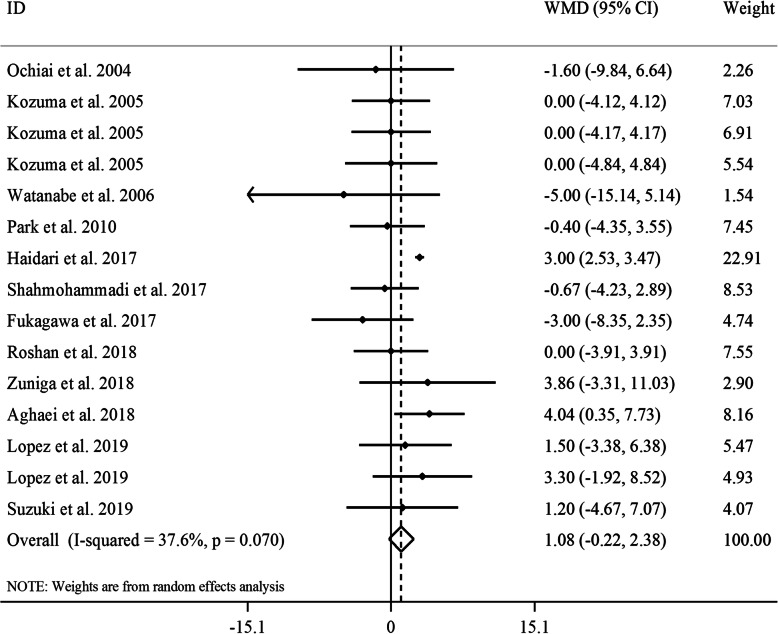
Forest plot for the effect of GCE supplementation on serum HDL concentrations, expressed as mean differences between intervention and control groups. Horizontal lines represent 95% CIs. Diamonds represent pooled estimates from random-effects analysis. GCE: green coffee extract, HDL: high-density lipoprotein, WMD: weighted mean difference, CI: confidence interval

Totally, 12 studies provided data on the effect of GCE supplementation on serum HDL concentrations [17–24, 26–29]. Combining 15 effect sizes from these studies revealed no significant effect of GCE supplementation on HDL levels (WMD: 1.08, 95% CI: − 0.22, 2.38 mg/dL, *P* = 0.10) (Fig. 6). A marginally significant between-study heterogeneity was seen in this regard (*I*^*2*^: 47.6, *P* = 0.02). In the subgroup analyses, we found that gender, length of follow-up, baseline levels of HDL and participants’ compliance were potential sources of heterogeneity. In addition, GCE supplementation resulted in a significant increase in serum HDL concentrations in trials included only female subjects, studies with ≥8 weeks of intervention duration, studies that were performed on participants with low levels of baseline serum HDL and studies with low or unclear adherence of participants to intervention. The sensitivity analysis revealed that the overall estimate depended on two studies [18, 26]. When we excluded these studies, Shahmohammadi et al. (WMD: 1.32, 95% CI: 0.03, 2.62 mg/dL, *P* = 0.04) and Fukagawa et al. studies (WMD: 1.44, 95% CI: 0.23, 2.64 mg/dL, *P* = 0.02), a significant increasing effect of GCE supplementation on serum HDL concentrations was observed. No evidence of a substantial publication bias was seen according to the Begg’s test (*P* = 0.80) and funnel plot (Supplemental Figure 2F).

In the dose-response analysis, we found a significant non-linear association between CGA dosage and serum concentrations of HDL (P_non-linearity_ = 0.01); such that from dosage of 100 mg/day to higher levels, CGA administration had an increasing effect on serum HDL concentrations (Supplemental Figure 4D).

## Discussion

The role of coffee consumption in the risk of cardiovascular diseases has been debated for many years. Recent meta-analyses of prospective studies showed no potential health risk associated with the coffee intake [42, 43] even when heavily consumed [44]. Also, an umbrella review of meta-analyses revealed the protective effects of coffee consumption against cardiovascular risk factors [45]. Unlike observational studies, considerable controversy exists among clinical trials. In the current study, we summarized evidence from clinical trials investigating the effect of GCE supplementation on lipid profile and some glycemic indices. We found that GCE supplementation significantly reduced FBG and insulin levels. Considering the lipid profile, GCE supplementation resulted in a significant decrease in TC concentrations, but results from other lipid measures were not significant. However, GCE supplementation improved serum levels of TG, LDL, and HDL in some subgroups of studies. Also, in the non-linear dose-response analyses, we found that the associations between CGA dosage and serum levels of FBS, TG, and HDL were in a non-linear fashion.

We found a significant reduction in both serum levels of FBG and insulin following GCE supplementation. In line with our findings, Morvaridi et al. reported a beneficial effect of green coffee consumption on glycemic indices and cardio-metabolic risk factors in adults [46]. In contrast with our findings, in a meta-analysis, Kondo et al. reported no significant effect of caffeinated/decaffeinated coffee consumption on FBS and insulin levels [47]. The observed controversy in this regard might be due to the use of different types of coffee across previous clinical trials. Green coffee contains a higher amount of CGA compared with other types of coffee. It seems that the anti-diabetic effect of green coffee is attributed to its CGA content. As seen in the dose-response analysis, the stronger reducing effect of GCE on FBG was observed at CGA dosage of 200 mg/d or more. CGA increases peripheral glucose disposal through activating AMP-activated protein kinase (AMPK) [48]. It also reduces glucose production by gluconeogenesis and glycogenolysis through inhibiting glucose-6-phosphatase [49]. Moreover, increased serum concentrations of adiponectin, a protein hormone released from adipose tissue that modulates glucose regulation and fatty acid oxidation, may play a role in the metabolic effect of CGA [50]. Reducing blood glucose through the mentioned pathways can also decrease insulin levels.

In the overall meta-analysis of the effect of GCE on lipid profile, we found a reducing effect only on serum levels of TC. In agreement with our findings, a prospective cohort study indicated a significant inverse association between coffee consumption, particularly green coffee, and TC levels [51]. Also, in an experimental study, GCE administration could significantly reduce TC levels [52]. In contrast, available findings on the effects of other types of coffee on lipid profile are conflicting [30]. Different findings might be explained by different processing methods used for preparation of coffee. In a systematic review, Penson et al. reported that the type of coffee and the methods of preparation are important for the effect of coffee consumption on serum levels of lipoproteins [53]. In addition, different duration of intervention, recruiting participants with different health conditions and different quality of clinical trials are other reasons for the observed discrepancy across clinical trials investigating the effects of coffee consumption on TC levels.

Although the overall analysis did not show significant effects of GCE supplementation on serum levels of LDL and HDL, we found favorable effects in studies that recruited female subjects. Further, the TC-lowering effect increased in studies conducted on either gender or female subjects. The analysis also showed a significant reducing effect of GCE on serum levels of TG and FBG among studies that included both sexes. It seems that sex may mediate the effect of GCE on lipid profile with a stronger effect observed in women. It has been shown that CGA is more durable in women than men and therefore, had a longer effect on women than men [54]. Also, the sex differences may be mediated by changes in steroid hormone levels [55].

In our meta-analysis, the beneficial effects of GCE supplementation were mostly observed in RCTs with a long duration of the intervention (≥8 weeks). It is consistent with the results from the previous meta-analysis on green coffee consumption in which a longer duration (> 8 weeks) was more effective on lipid profile [56]. In a network meta-analysis, Kondo et al. concluded that clinical trials with a longer duration of intervention can better clarify the potential effects of coffee [47]. Thus, green coffee consumption should be long enough to improve the lipid profile.

Dose-response analysis demonstrated a significant increment in serum levels of HDL from CGA dosage of 100 mg/day to higher amounts. Moreover, we found a significant non-linear association between CGA dosage and serum TG concentrations. However, the TG-lowering effect of CGA was decreased at a dosage of 500 mg/d and over. The effect of green coffee on lipid profile was also dose-dependent in earlier meta-analyses of RCTs [56, 57]. Therefore, the dosage of CGA is a potential moderator for the beneficial effect of GCE on lipid profile. CGA may exert its lipid-lowering effects through inhibition of the lipids absorption and the formation of cholesterol micelles. Also, CGA is involved in modifying hepatic metabolism of cholesterol and fatty acids by inhibiting pancreatic lipase and hydroxymethyl glutaryl Co-A reductase and increasing the activity of fatty acid beta-oxidation and the expression of peroxisome proliferator-activated receptor-alpha in the liver [13, 58]. Based on our finding, however, higher dosages of more than 500 mg/d CGA is not recommended. Of note that higher intake of CGA may elevate homocysteine levels, a risk factor for cardiovascular disease, and stimulate the release of adrenaline generating several effects on the cardiovascular system including increased blood pressure and reduced insulin sensitivity [50, 59, 60].

The current meta-analysis was the first to summarize available findings on the effects of GCE supplementation on glycemic and lipid profiles. The selected studies were from different countries which increase the generalization of findings to the various ethnic groups. Other strengths of this meta-analysis were the inclusion of all clinical trials written in all languages, lack of publication bias, and moderate-to-high quality of the most included studies. However, some limitations should be considered when interpreting our findings. These limitations include the lack of evaluation of participants’ compliance in a limited number of RCTs, the lack of controlling for baseline values of glycemic and lipid measures in some others, short duration of intervention in some trials, and different health conditions of participants across included studies. Also, we could not find the effect of GCE supplementation on other glycemic indices such as HbA1c due to limited number of studies.

## Conclusion

GCE supplementation had favorable effects on glycemic indices including FBG and insulin levels. In terms of lipid profile, GCE supplementation led to a significant reduction in serum TC, particularly in individuals with elevated levels of TC. This suggests GCE as a promising antihyperlipidemic agent since some patients do not achieve cholesterol reduction goals or cannot tolerate statins due to adverse effects [61]. We also found a significant favorable effect of GCE on serum levels of TG, LDL, and HDL in some subgroups. The effect was more prominent in women and studies with a long duration of intervention. Further studies are required to find the effect of GCE supplementation on patient-reported outcomes including quality of life, as well as the effect of genetic polymorphisms on the pharmacokinetics of CGA to explain the interindividual variability.

## Supplementary information

**Additional file 1: Supplemental Table 1**. Results of risk of bias assessment for clinical trials included in the current meta-analysis on the effects of GCE supplementation on glycemic and lipid measures^1^. **Supplemental Figure 1.** Flow diagram of study selection. **Supplemental Figure 2.** Funnel plots for the effect of GCE supplementation on serum levels of FBG (A), insulin (B), TG (C), TC (D), LDL (E), and HDL (F). WMD: weighted mean difference, FBG: fasting blood glucose, TG: triglyceride, LDL: low-density lipoprotein, HDL: high-density lipoprotein. **Supplemental Figure 3**. Non-linear dose-response effects of CGA dosage (mg/d) on (A) FBG and (B) serum levels of insulin. The 95% CI is demonstrated in the shaded regions. CGA: chlorogenic acid, FBG: fasting blood glucose. **Supplemental Figure4**. Non-linear dose-response effects of CGA dosage (mg/d) on serum concentrations of (A) TG, (B) TC, (C) LDL, and (D) HDL. The 95% CI is demonstrated in the shaded regions. CGA: chlorogenic acid, TG: triglycerides, TC: total cholesterol, LDL: low-density lipoprotein, HDL: high-density lipoprotein

## Data Availability

The datasets analyzed during the current study are available from the corresponding author on reasonable request.
