# Supplementary material for: The effects of green coffee extract supplementation on glycemic indices and lipid profile in adults: a systematic review and dose-response meta-analysis of clinical trials
Source: Nutr J. 2020 Jul 14;19:71. doi: 10.1186/s12937-020-00587-z (PMC7362645; doi:10.1186/s12937-020-00587-z)
Supplement: Supplementary file 1 — Additional file 1: Supplemental Table 1. Results of risk of bias assessment for clinical trials included in the current meta-analysis on the effects of GCE supplementation on glycemic and lipid measures1. Supplemental Figure 1. Flow diagram of study selection. Supplemental Figure 2. Funnel plots for the effect of GCE supplementation on serum levels of FBG (A), insulin (B), TG (C), TC (D), LDL (E), and HDL (F). WMD: weighted mean difference, FBG: fasting blood glucose, TG: triglyceride, LDL: low-density lipoprotein, HDL: high-density lipoprotein. Supplemental Figure 3. Non-linear dose-response effects of CGA dosage (mg/d) on (A) FBG and (B) serum levels of insulin. The 95% CI is demonstrated in the shaded regions. CGA: chlorogenic acid, FBG: fasting blood glucose. Supplemental Figure4. Non-linear dose-response effects of CGA dosage (mg/d) on serum concentrations of (A) TG, (B) TC, (C) LDL, and (D) HDL. The 95% CI is demonstrated in the shaded regions. CGA: chlorogenic acid, TG: triglycerides, TC: total cholesterol, LDL: low-density lipoprotein, HDL: high-density lipoprotein [file 12937_2020_587_MOESM1_ESM.docx]

**Online Supplementary File**

**Supplemental Table 1**: Results of risk of bias assessment for clinical trials included in the current meta-analysis on the effects of GCE supplementation on glycemic and lipid measures^1^

| Study | Random Sequence Generation | Allocation concealment | Blinding of participants personnel | Blinding of outcome assessors | Incomplete outcome data | Selective outcome reporting | Other sources of bias |
| --- | --- | --- | --- | --- | --- | --- | --- |
| Aghaei et al. 2018 | L | H | L | H | L | H | L |
| Fukagawa et al. 2017 | L | U | L | L | L | H | L |
| Haidari et al. 2017 | L | U | L | L | L | U | L |
| Kim et al. 2012 | L | U | L | H | L | H | L |
| Kozuma et al. 2005 | L | U | L | L | L | U | L |
| Lopez et al. 2019 | L | H | H | L | L | H | L |
| Ochiai et al. 2004 | H | U | L | H | H | U | L |
| Park et al. 2010 | L | U | L | L | L | U | L |
| Roshan et al. 2018 | L | L | L | L | L | L | L |
| Sarria et al. 2018 | L | H | H | L | L | H | L |
| Shahmohammadi et al. 2017 | L | L | L | L | L | U | L |
| Suzuki et al. 2019 | H | U | L | H | L | H | L |
| Watanabe et al. 2006 | L | U | H | H | L | U | L |
| Zuniga et al. 2018 | L | H | L | H | L | U | L |

^1^Each study was assessed for risk of bias using the Cochrane Risk of Bias Assessment tool (Ref. 32).

**Online Supplementary File**


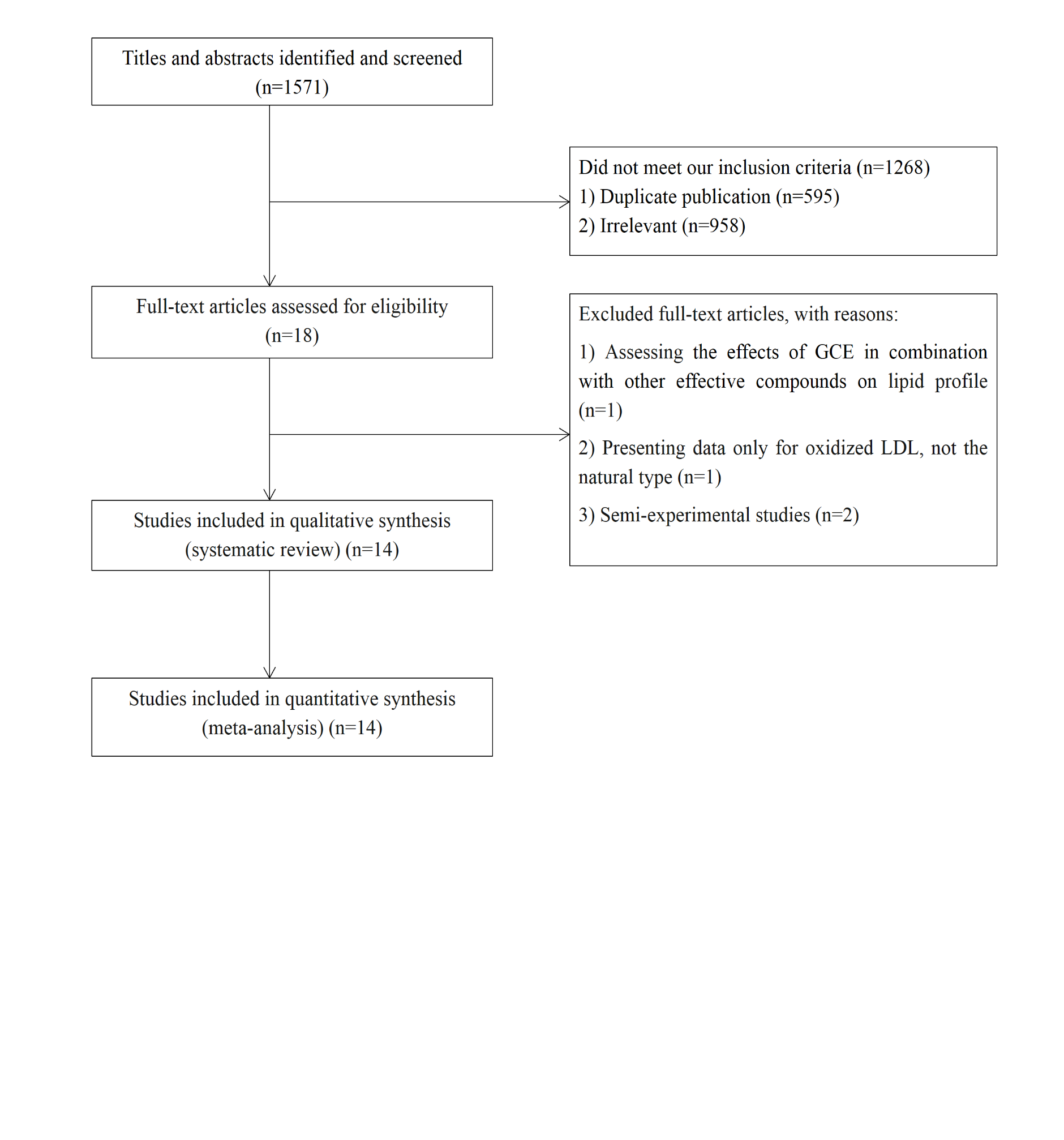


**Supplemental Figure 1:** Flow diagram of study selection

**Online Supplementary File**


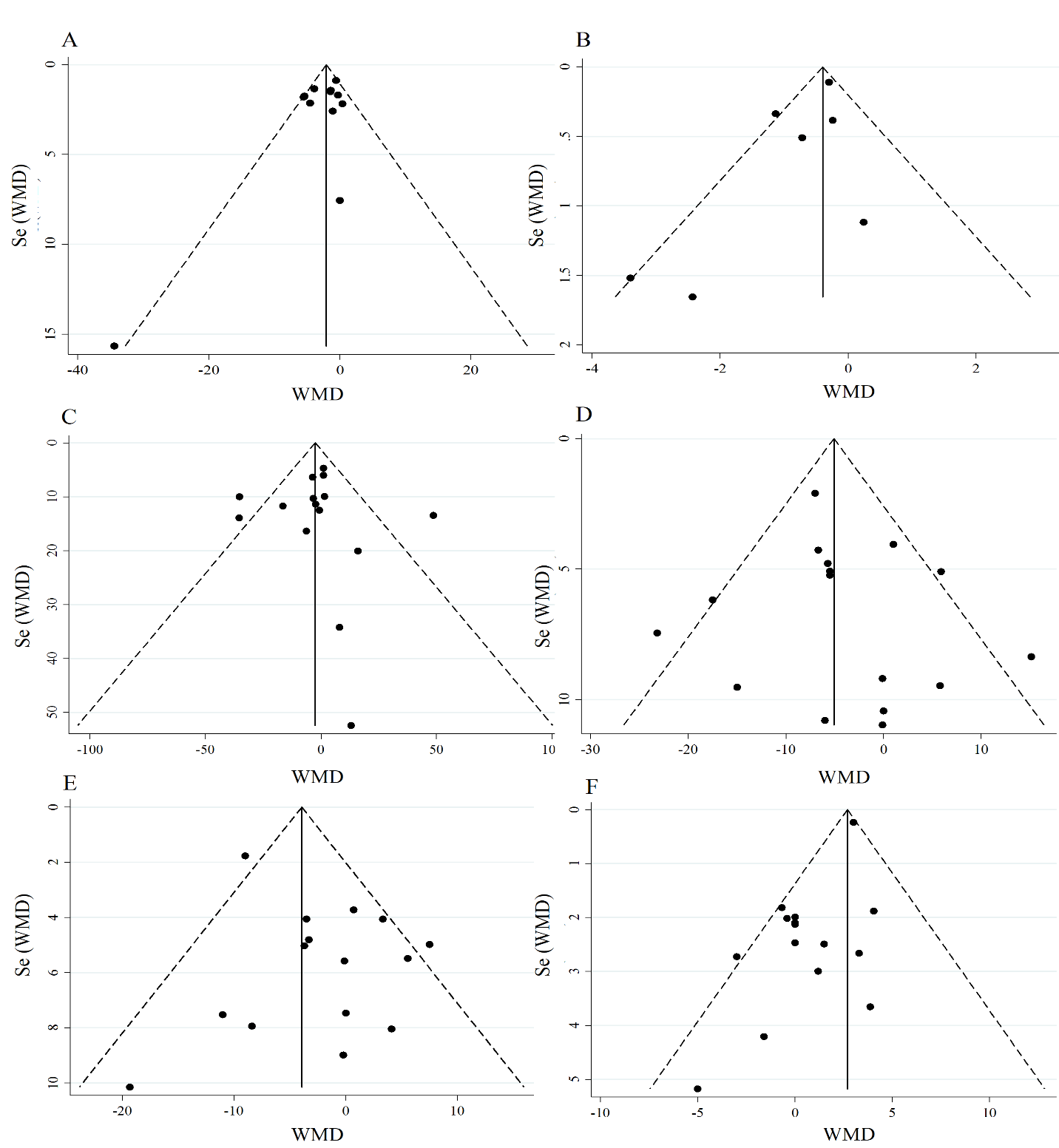


**Supplemental Figure 2:** Funnel plots for the effect of GCE supplementation on serum levels of FBG (A), insulin (B), TG (C), TC (D), LDL (E), and HDL (F). WMD: weighted mean difference, FBG: fasting blood glucose, TG: triglyceride, LDL: low-density lipoprotein, HDL: high-density lipoprotein

**Online Supplementary File**


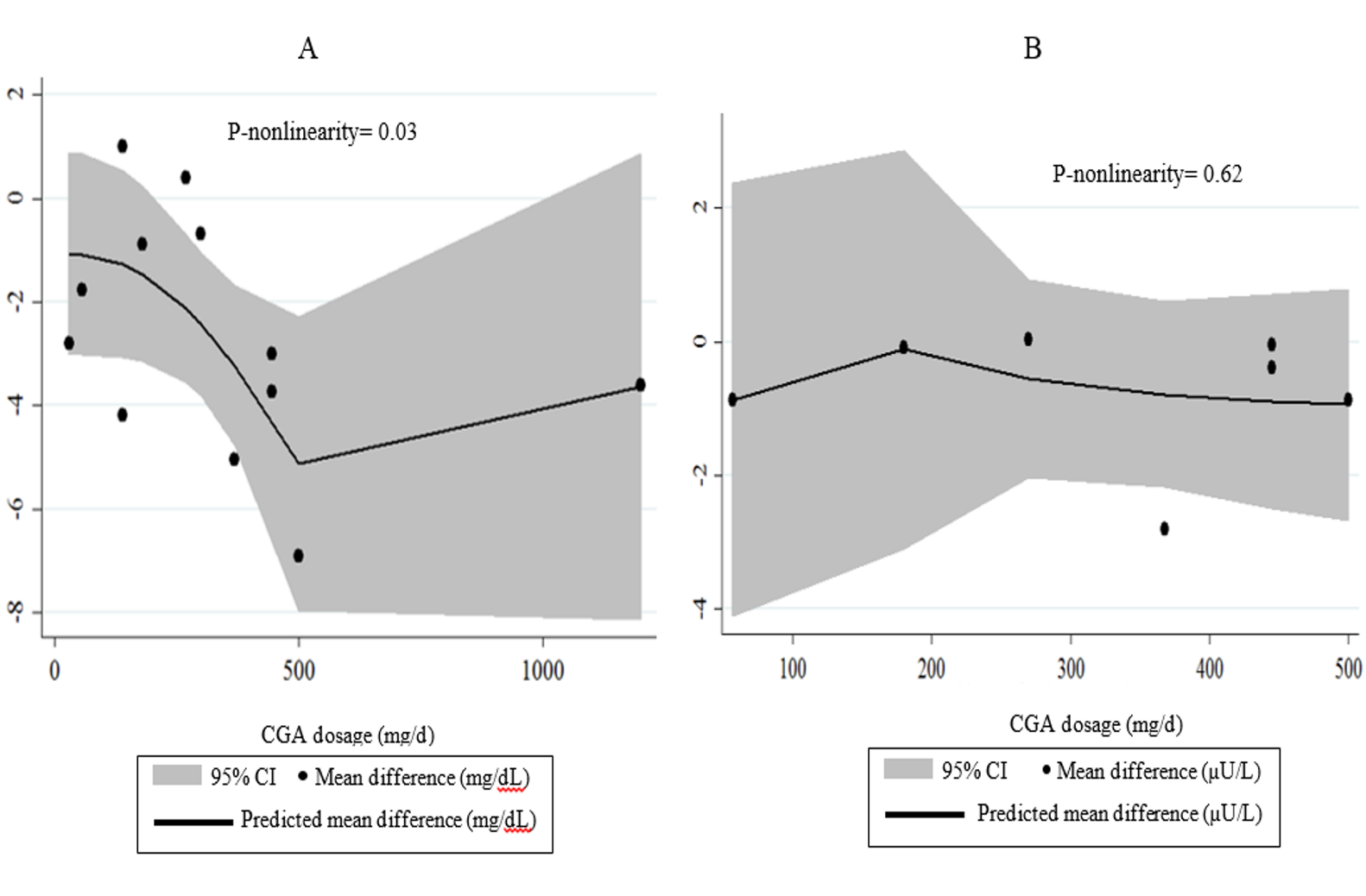


**Supplemental Figure 3**: Non-linear dose-response effects of CGA dosage (mg/d) on (A) FBG and (B) serum levels of insulin. The 95% CI is demonstrated in the shaded regions. CGA: chlorogenic acid, FBG: fasting blood glucose

**Online Supplementary File**


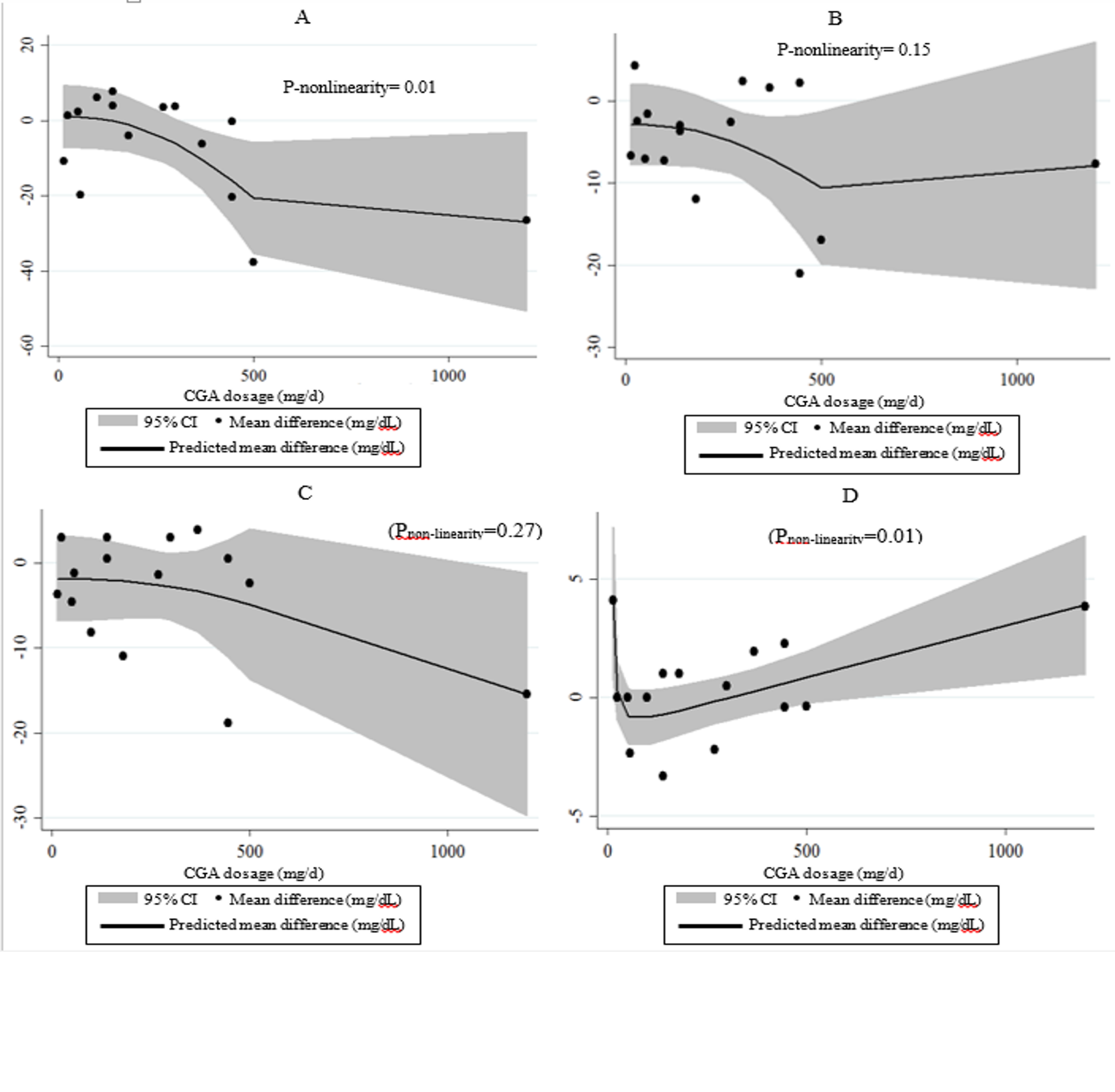


**Supplemental Figure 4**: Non-linear dose-response effects of CGA dosage (mg/d) on serum concentrations of (A) TG, (B) TC, (C) LDL and (D) HDL. The 95% CI is demonstrated in the shaded regions. CGA: chlorogenic acid, TG: triglycerides, TC: total cholesterol, LDL: low-density lipoprotein, HDL: high-density lipoprotein
